# Training trial of critical care paramedics for non-medical authorisation of blood

**DOI:** 10.29045/14784726.2022.03.6.4.55

**Published:** 2022-03-01

**Authors:** Hazel Smith, Heidi Doughty

**Affiliations:** NIHR Surgical Reconstruction and Microbiology Research Centre; NIHR Surgical Reconstruction and Microbiology Research Centre; NHS Blood and Transplant

**Keywords:** NMA, paramedics, pre-hospital transfusion, training

## Abstract

The use of pre-hospital blood transfusion by air ambulance crews is increasing. Blood transfusion is traditionally ‘authorised’ by doctors, not prescribed. However, there is an increasing interest in extending the capability of authorisation to other practitioners – that is, non-medical authorisation (NMA). A UK framework for nurses and midwives has existed since 2007, but training for critical care paramedics (CCPs) has been limited.

The Resuscitation with Pre-Hospital Blood Products (RePHILL) trial was launched in 2016, requiring pre-hospital administration of red cells and LyoPlas. Authorisation was initially restricted to doctors, leading to missed recruitment by paramedic-only crews. The trial protocol was amended in 2019 to permit NMA following suitable training and stakeholder consultation. We present a targeted training programme designed to support paramedic-led transfusion within the framework of the pre-hospital trial.

We considered the knowledge and skills required for NMA and compared this with baseline knowledge from paramedic training to identify the training gap. We examined examples of existing military and civilian NMA training to develop a targeted programme for a single air ambulance. The four elements of our training programme were pre-course online training, previous trial participation, face-to-face training and competency assessment.

Training was delivered to three CCPs, who cascaded the training to 14 colleagues. The training time was one morning, including a face-to-face session and assessment. Novel topics included physiological triggers for transfusion and transfusion risks in the pre-hospital environment. Paramedics were encouraged to recognise and report new patterns of adverse events. Reflective feedback suggests the programme provided CCPs the knowledge to autonomously recruit trial patients and authorise transfusion.

## Introduction

The use of pre-hospital blood transfusion by air ambulance crews is increasing in England and Europe (Thies et al., 2020). Blood is traditionally authorised by doctors, not prescribed. However, there is increasing interest in extending the capability to other practitioners – that is, non-medical authorisation (NMA). A UK framework for nurses and midwives has existed since 2007 (Pirie & Sinclair, 2013) and has provided a firm foundation for training and governance. Access to NMA training for critical care paramedics (CCPs) has been limited, although in Wales training was extended to CCPs in September 2017.

The Resuscitation with Pre-Hospital Blood Products (RePHILL) trial was launched in 2016. The trial compared standard saline resuscitation in major traumatic haemorrhage with red cells and freeze-dried plasma (Smith et al., 2017). The trial required pre-hospital authorisation for the use of blood; however, until April 2019 this role was restricted to doctors, leading to missed recruitment by paramedic-led pre-hospital emergency medical (PHEM) responses. Following supportive advice from the Health and Care Professions Council (HCPC) and the Medicines and Healthcare products Regulatory Authority (MHRA), an amended trial protocol allowed suitably trained paramedics to confirm eligibility and authorise transfusion. We present our experience of a targeted training programme designed to support paramedic-led transfusion within the framework of a pre-hospital trial.

## Methods

We considered the NMA knowledge and skills required for the pre-hospital trial against the baseline knowledge from CCP and trial training to identify the transfusion training gap. We then examined examples of military and civilian NMA training to develop a targeted programme sponsored by the PHEM clinical lead for The Air Ambulance Service (TAAS).

### Paramedic clinical training

Paramedic study includes the physiology and management of shock together with the basics of haematology. Some courses include transfusion competency arranged with a local hospital trust, using standards defined by the 2006 Patient Safety Notice (National Patient Safety Agency, 2006). TAAS CCPs have a Post-Graduate Certificate in Pre-Hospital Care which enables them to provide more specialised care. This includes administration of drugs such as midazolam and ketamine, plus advanced skills such as thoracostomy. In addition, clinical support for the RePHILL trial had provided exposure to the equipment and processes involved in pre-hospital transfusion.

### Trial training

All our trial CCPs had already undertaken good clinical practice (GCP) and trial-specific training for RePHILL. GCP ensures that ‘everyone involved in research is trained or appropriately experienced to perform the specific tasks they are being asked to undertake’ (National Institute for Health Research [NIHR], n.d.). Trial-specific training included resuscitation treatment algorithms, preparation of LyoPlas and adverse reporting procedures.

### Existing non-medical authorisation courses

Civilian courses reviewed included the well-established NHS Blood and Transplant (NHSBT) course (NHSBT, 2017) and a hospital-based course in the East Midlands. Both were originally designed for nursing. Military courses for paramedics included bespoke packages as well as training available through the Norwegian-based Trauma Haemostasis and Oxygenation Research (THOR) network (THOR Network, 2020). Hospital-based courses focus on decision-making and appropriate component use underpinned by haematological principles in different clinical scenarios. We judged that some of these elements were less relevant when using ‘universal’ O negative red cells and group A/AB lyophilised plasma within a pre-hospital trial framework. The THOR programme is physiology and trauma focused. However, it is currently based on whole blood, which is not yet available in the United Kingdom outside of studies. Despite these limitations, elements of each of these programmes were integrated into a short pre-hospital transfusion training programme for CCPs.

### NMA training programme

We designed a classroom-based session and assessment to address the role requirements and complement earlier training. The programme included:

1. an introduction to the background of NMA and the use of LyoPlas;
2. the RePHILL trial, physiology of shock and decision to transfuse; and
3. recognising, managing and reporting pre-hospital hazards of transfusion.

**Figure 1:**
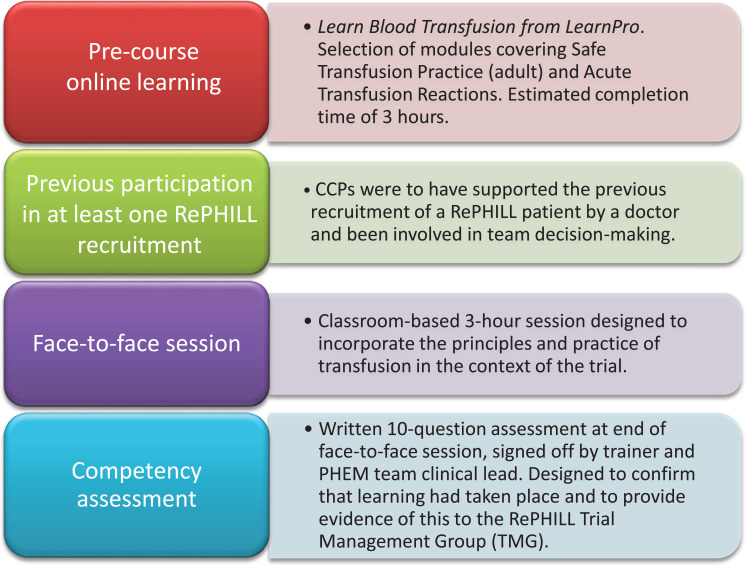
Figure 1. Non-medical authorisation training programme for trial participants.

TAAS requested cascade training starting with a face-to-face session for three CCPs at a local hospital with a history of teaching NMA. The training team consisted of a consultant anaesthetist with specialist interest in transfusion, a transfusion practitioner and the RePHILL research paramedic. All three were involved in designing the programme, together with a consultant in transfusion medicine with experience in military and civilian NMA. The four elements of our training programme are shown in Figure 1.

### Feedback

Students were invited to supply immediate feedback via email at the end of the session. Following notification via the trial database of a patient recruited by a CCP, the member of staff was invited to reflect on their experience. Both of these were in the form of unstructured feedback in order to allow for as much freedom of expression as possible.

## Results

### Training feedback

Face-to-face training of three CCPs took place in December 2019. Post-course feedback was positive and reflected the importance of all elements of the package.

Initially participants confessed to feeling a little unsure before attending:

Prior to the session I had completed the online transfusion training [United Kingdom and Republic of Ireland Blood Transfusion Network, 2018], however I knew there were still areas I was unsure of – including ABO blood types and the associated risks of incorrectly administering blood to the wrong patient. (CCP1)

Prior to attending the session I was concerned that the level of knowledge I had regarding the management of any transfusion-related complications was insufficient and/or that my general knowledge about the more theoretical aspects of blood transfusion would be insufficient. (CCP2)

We were pleased to hear that we had allayed these fears and provided them with an improved level of confidence:

The unique environment critical care paramedics work in creates challenges in identifying adverse transfusion reactions, but the session covered them, with practical advice and solutions. Areas such as ABO blood types were also discussed but reassurances given that . . . limited exposure to blood products meant concerns were alleviated. (CCP1)

I had convinced myself that spotting these reactions was somewhat of a dark art, they were things that I must by hypervigilant for. The reassurance that any reaction was likely to look like a normal anaphylaxis and that the treatment was the same has allowed me to relax a little. (CCP2)

In addition, participants were keen to pass on this knowledge and enthusiasm and subsequently trained all 14 of their CCP colleagues within three months. There were surprises in the feedback, too. CCP2 stated:

Going forward I see no reason that CCP-led administration within the RePHILL trial will be in any way different to enrolments made when a doctor is on board. Indeed, I feel it may be slightly more straightforward as we are perhaps more used to following the protocol and completing the required documentation.

These comments support the authors’ argument that paramedics are already ideally placed to confirm eligibility for a trial. Firstly, trial inclusion and exclusion criteria mirror the structure of drug indications and contra-indications within the Joint Royal Colleges Ambulance Liaison Committee (JRCALC) guidelines (Association of Ambulance Chief Executives, 2021) which paramedics are used to referring to daily. Secondly, paramedics are autonomous, specialist practitioners accustomed to accommodating an expanding scope of practice.

### CCP recruitment in practice

During the first ‘live’ case, a RePHILL doctor was present but left the actual enrolment to CCP3. This allowed the CCP to autonomously undertake the enrolment but with the RePHILL trained doctor available on scene for support should he have needed it. The CCP’s feedback showed how the training had boosted confidence:

The training I received led to a slick and proficient RePHILL recruitment, and I was left feeling the patient had received the best possible care. Furthermore, had there been any adverse incidents I felt I would have had the knowledge to manage them appropriately. (CCP3)

The second paramedic-led recruitment supports the reason for NMA training. The PHEM doctor had not yet completed trial training; therefore the opportunity to recruit this patient would have been missed. Once again feedback from the CCP involved supported the value of his NMA training:

I won’t deny that I was probably a little rusty with it being the first enrolment I’d done post-pandemic, but I still felt fairly confident in carrying out the process, which is testament to the previous training provided. (CCP4)

He also highlighted the importance of continued learning following the initial training:

Moreover, as full-time CCPs, it has become apparent that our continued exposure and experience of enrolling patients to the trial has been beneficial to many of our doctor colleagues who do far fewer PHEM shifts and are less familiar with the logistics and processes involved. (CCP4)

This comment highlighted something we had not considered when developing the training package. Having all clinical members of staff on scene trained in NMA ensures that mutual support can be provided. Sometimes it is not clinical grade or level of knowledge that is important; exposure and experience are equally valid.

## Discussion

The growing appreciation of early transfusion-based resuscitation has led to a considerable interest in pre-hospital transfusion. The UK paramedic community are well placed to support both trials and transfusion (Doughty & Naumann, 2020). However, few have received NMA training; neither have the specific requirements of this training been decided. We have presented the design and initial feedback from a small, bespoke training programme. Training was well received by those involved, whether received via face-to-face or cascade. The first paramedic-led recruitment was successfully delivered three months after the training. Further consolidation in practice has been disrupted by the infrequent nature of recruitment and the COVID-19 crisis.

The civilian paramedic literature suggests their role in pre-hospital transfusion is rarely formally published. However, the increasing interest in early blood-based resuscitation is stimulating an expanding scope of practice in both civilian and military practice. And there is a growing body of evidence to show that it is both safe and effective. For example, in a retrospective analysis of 136 trauma patient records between 2011 and 2015, an Australian study investigated paramedic blood authorisation and found that, in cases reviewed by a doctor, 90% of all decisions made by paramedics to give blood were appropriate (Heschl et al., 2018).

Established military programmes include pre-hospital plasma-only resuscitation using LyoPlas successfully introduced in 2011 by the Israeli Defence Force (Shlaifer et al., 2017) and more recently the US military selected ‘first responder’ programmes using both freeze-dried plasma and group O whole blood (Fisher et al., 2015).

During the RePHILL trial there was no UK commercially available NMA course suitable for our paramedics. We therefore devised our short course primarily using material from the military-civilian THOR network which promotes pre-hospital transfusion. Their Blood Far Forward programme is designed primarily for the Austere Medical Environment (Strandenes et al., 2013). THOR field courses train paramedics and others to both collect and administer whole blood and give lyophilised plasma. The underpinning knowledge emphasises shock physiology and associated ‘blood failure’ (White et al., 2017). Training sessions over several days supplied both theory and high-fidelity role rehearsal. The breadth and depth of training was more than needed for our purpose but highlights a different emphasis for education and training.

As the paramedic scope of practice continues to develop, they are increasingly involved in trials. Paramedics are uniquely placed to contribute to both the evidence base and practice development in pre-hospital transfusion. The pre-hospital environment is challenging both clinically and logistically and presents novel risks in transfusion practice. Consequently, we propose that new patterns of adverse events should be identified by all members of the pre-hospital team and reported to the national haemovigilance scheme, Serious Hazards of Transfusion (SHOT), to inform future policy and practice.

### Limitations

Our training programme was developed for a specific trial and should not be currently used outside of that framework. However, the programme and experience serve to inform development of NMA courses for the wider pre-hospital community.

## Conclusion

We present the first structured course of its type aimed specifically at the paramedic profession in the context of a trial. The feedback suggests the programme provided CCPs the knowledge to autonomously recruit trial patients and authorise transfusion. Pre-hospital transfusion continues to develop, and we propose that paramedics have an important role in this. In addition, NMA courses should recognise the unique challenges of the pre-hospital environment.

## Acknowledgements

We are indebted to Dr Caroline Leech and The Air Ambulance Service team for spear-heading NMA within the RePHILL trial. We thank Dr Falguni Chocksey, Consultant Anaesthetist and Sarah Aston, Transfusion Practitioner from University Hospitals Coventry and Warwickshire NHS Trust for providing bespoke face-to-face training and for their support to the CCPs and the project.

## Author contributions

HS and HD designed the training concept and drafted the manuscript. All students cited have provided permission to directly quote from their reflective notes. HS acts as the guarantor for this article.

## Conflict of interest

None declared.

## Funding

HS is funded by the NIHR EME programme. EudraCT No.: 2015-001401-13. This report presents independent research funded by the National Institute for Health Research (NIHR). The views expressed are those of the author(s) and not necessarily those of the NHS, the NIHR or the Department of Health and Social Care.
